# Pirfenidone alleviates smoke inhalation lung injury of rats via the NF‐κB signaling pathway

**DOI:** 10.1002/iid3.70014

**Published:** 2024-11-26

**Authors:** Tingting Lv, Kaiyuan Yang, Jinxiang Wang

**Affiliations:** ^1^ Department of Respiratory and Critical Care Medicine, Beijing Luhe Hospital Capital Medical University Beijing China

**Keywords:** apoptosis, inflammation, oxidative stress, PFD, SILI

## Abstract

**Objective:**

Smoke inhalation lung injury (SILI) is a common complication in fires and wars, characterized by acute onset and severe condition. Pirfenidone (PFD), a new small‐molecule drug, has been shown to improve lung function and inhibit pulmonary fibrosis and inflammation. This study aimed to elucidate the effect and underlying mechanism of PFD on SILI in rats.

**Materials and Methods:**

SILI rats were constructed using a homemade smoking device, which was then treated with PFD. The blood was collected from the abdominal aorta, and the arterial blood gas was detected. The productions of oxidative stress markers and inflammatory cytokines in plasma were measured by enzyme linked immunosorbent assay assay. Moreover, the alveolar surface area, wet:dry weight ratio of the lung tissues, and bronchoalveolar lavage fluid (BALF) were determined as well. The pulmonary histopathology, cell apoptosis, and the related proteins of nuclear factor kappa B (NF‐κB) pathway were determined by hematoxylin‐eosin staining, TdT‐mediated dUTP‐biotin nick end labeling, and western blot assays, respectively.

**Results:**

PFD had a significant protective effect on SILI via inhibiting oxidative stress, inflammation, and apoptosis. Mechanistically, PFD inhibited the activation of NF‐κB pathway in vivo. Moreover, activation of NF‐κB pathway attenuated the PFD‐mediated protective effect against SILI.

**Conclusions:**

These data demonstrate that PFD alleviates SILI of rats via the NF‐κB signaling pathway, which provides an attractive therapeutic option for SILI treatment.

## 1 INTRODUCTION

Smoke inhalation lung injury (SILI) refers to the injury of airway or lung parenchyma caused by a large number of toxic gases and various particles entering the respiratory tract, with a case fatality rate of up to 60%.^1^, ^2^ The pathophysiological mechanism of SILI is very complex, with inflammation and oxidative stress being one of the main causes.^3^, ^4^ Numerous of studies have confirmed that smoke inhalation activates lung macrophages, neutrophils, endothelial cells, and vascular smooth muscle cells to release a large number of inflammatory cytokines, such as tumor necrosis factor‐α (TNF‐α), interleukin‐1β (IL‐1β), and IL‐6, which aggravate lung injury.^5^ In addition, nitric oxide (NO), nitrous oxide (N_2_O) and sulfur dioxide (SO_2_) contained in smoke enter the body as oxidants to stimulate granulocytes to produce excessive reactive oxygen species (ROS) and malondialdehyde (MDA), which aggravate oxidative stress‐induced lung injury. Superoxide dismutase (SOD) and glutathione peroxidase (GSH‐Px) are important antioxidant enzymes, which protect cells from oxidant‐induced damage. At present, the main treatment for SILI patients are mechanical ventilation and liquid resuscitation, but long‐term mechanical ventilation aggravates lung injury, while liquid therapy leads to different degrees of damage to other organs,^6^ which is the main reason for the high mortality and poor prognosis of SILI patients. Therefore, it is urgent to search for more effective treatment for SILI.

Pirfenidone (5‐methyl‐1‐phenyl‐2‐(1H)‐pyridone, PFD) is an oral pyridone analogue that is approved for the treatment of idiopathic pulmonary fibrosis and slowing down lung function decline.^7^, ^8^ It has been reported that PFD plays an important role in kidney disease, hypertrophic cardiomyopathy, rheumatoid arthritis, and malignancy.^9^, ^10^, ^11^, ^12^ A previous study has shown that PFD inhibits the production of inflammatory cytokines, such as IL‐6, IL‐17, and TNF‐α.^13^ Another study demonstrated that PFD has anti‐fibrotic, anti‐inflammatory, and antioxidant activity in progressive fibrotic and inflammatory diseases.^14^, ^15^ Notably, one study suggests that PFD has a protective effect on acute lung injury induced by paraquat in rats.^16^ Moreover, PFD has been shown to reduce LPS‐induced acute lung injury.^17^ However, the therapeutic effects and underlying mechanisms of PFD in SILI remains incompletely understood.

Nuclear factor kappa B (NF‐κB) is a major inflammatory regulator that is activated in response to inflammatory stimulation. Tsuchiya et al.^18^ discovered that PFD therapy mitigated endotoxin‐induced liver injury and hepatocyte apoptosis by inhibiting the activation of NF‐κB pathway. El‐Agamy et al.^19^ showed that PFD improved concanavalin A‐induced hepatitis in mice by modulating the NF‐κB pathway. In addition, the drug mechanism by which PFD exhibits the protective effects on pulmonary fibrosis in rats through inhibiting the NF‐κB and MAPK signaling pathway has become a hot research topic worldwide abroad.^20^

The present study aims to investigate the therapeutic effects and underlying mechanisms of PFD in SILI. A smoke generator was applied to construct SILI models in rats, and the therapeutic effects of PFD on oxidative stress, inflammation, and apoptosis in SILI rats were investigated. Results demonstrated that the NF‐κB pathway was involved in the anti‐inflammatory and antiapoptotic effects of PFD in SILI.

## 2 MATERIALS AND METHODS

### 2.1 Animals

A total of 50 healthy male Sprague‐Dawley (SD) rats, aged 8–10 weeks, with a body weight of (180 ± 20) g, were procured from Beijing Academy of Military Medical Sciences (Beijing, China) and kept in cages at the Animal Center of Capital Medical University. The feeding environment was specific pathogen free level, the ambient temperature was 22°C–26°C, the humidity was 40%–60%, the light/dark environment was 12 h cycle, and the rats were free to eat and water. After feeding for 7 days, it was used for follow‐up experiment. The experiment was reviewed by the Ethics Committee of Capital Medical University (No. AEEI‐2023‐156), and all animal experiments followed the feeding and use standards of experimental animals formulated by the National Institute of Health Research.

### 2.2 Construction of SILI model in SD rats

A self‐made smoke generator was used to construct a model of SILI in SD rats as previously described.^21^ In brief, the SD rats were placed in a metal squirrel cage with an isolation mesh to separate them individually. The metal cage was then placed in a homemade smoke generator (150 g/kg [sawdust/body weight]). The total exposure time to smoke was 3 min, which was divided into 4 exposures (45 s each time). After 45 s of each smoke exposure, the cage was removed from the smoke storage chamber into the ambient air for 60 s to prevent suffocation of rats due to carbon monoxide poisoning. This cycle was repeated three times. After SILI for 5 min, the rats were fed under normal conditions. At 4 h post‐SILI, rats were randomly assigned to five groups: control, PFD, SILI, SILI + PFD, and SILI + PFD + phorbol myristate acetate (PMA, NF‐κB pathway agonist) groups. The rats in control group without smoke inhalation were given 1 mL 1% carboxymethyl cellulose (CMC) intraperitoneally once per day. The rats in PFD group received an intraperitoneal injection of 1 mL PFD (100 mg/kg) once per day.^22^ The rats in SILI group were given 1 mL 1% CMC intraperitoneally once per day after smoke inhalation. The rats in SILI + PFD group were given PFD (1 mL, 100 mg/kg, intraperitoneally, once per day) after smoke inhalation. The rats in SILI + PFD + PMA group were given PFD (1 mL, 100 mg/kg, intraperitoneally) and PMA (2 μg/kg, tail vein) once per day after smoke inhalation.^23^ After 3 days of continuous treatment, 2% pentobarbital sodium (50 mg/kg) was injected intraperitoneally for anesthesia, and the abdominal aortic blood was drawn after anesthesia. Then, the rats were killed and the lung lobes were taken for follow‐up experiments. The experimenters tried to avoid causing unnecessary pain to the rats while building the model.

### 2.3 Alveolar surface area measurement

The left lung tissue was cut into 5 μm sections after paraffin embedding. Five random visual fields were quantified using the pathology multimedia image analysis software Version2.0 (Olympus) by calculating the number of interceptions with the alveolar wall and determining where each line ended. The alveolar surface area is calculated as follows: Alveolar surface area = 4 × (Vp × Ialv)/(d × Pair); Vp = parenchymal volume, Ialv = number intercepts with alveolar septal walls, d = length of a single test line, and Pair = number of points hitting air spaces in alveoli and ducts.

### 2.4 Lung wet/dry (W/D) weight ratio

The W/D weight ratio was calculated to evaluate lung edema. In brief, the right lower lobe of lung tissue was excised, followed by irrigated with normal saline, and dried with filter paper. Then, the tissue was placed on a piece of aluminum foil and weighed immediately (wet weight). Afterwards, the wet tissue was dried in an oven at 60°C for 48 h and then re‐weighed (dry weight). The W/D weight ratio was calculated as wet weight/dry weight.^24^

### 2.5 Blood gas analysis

After anesthesia, 2 mL of abdominal aorta blood was taken from rats. Within 30 min, carbon dioxide partial pressure (PaCO_2_), oxygen partial pressure (PaO_2_), and oxygenation index (PaO_2_/FiO_2_ ratio) were measured by blood gas analyzer (Radiometer Medical, Copenhagen, Denmark).

### 2.6 Bronchoalveolar lavage fluid (BALF) analysis

After anesthesia and euthanasia, the anterior chest of the rats was cut, the left lung was separated and exposed, and the left main bronchus was ligation. Subsequently, 1 mL of sterile saline precooled at 4°C was injected into the left bronchus and aspirated three times. The aspirated BALF was collected and centrifuged at 1000 rpm at 4°C for 5 min to remove cell and tissue debris. The protein concentration of the supernatant was determined using the bicinchoninic acid (BCA) kit (Beyotime) according to the manufacturer's instructions.

### 2.7 Quantitative real‐time PCR (qRT‐PCR)

The RNeasy Plus Mini Kit (74134, QIAGEN) was applied to extract total messenger RNAs from lung tissues, and PrimeScript reagent Kit (Takara) was used for reverse transcription cDNA. QRT‐qPCR was performed on LightCycler480 Real‐Time PCR System (Roche) using SYBR Green PCR Master Mix (Takara). Glyceraldehyde‐3‐phosphate dehydrogenase (GAPDH) was used as an internal control reference. The relative expression of target genes was calculated by the 2^−ΔΔCt^ method.

### 2.8 Measurement of ROS level

The levels of ROS in lung tissues were measured using dichlorodihydrofluorescein diacetate (DCFH‐DA) staining (Sigma‐Aldrich) following the manufacturer's protocol. The cell precipitate of lung tissues was collected and then washed with phosphate buffer saline (PBS) for three times. Afterwards, the cells (1.0 × 10^3^ cells/well) were seeded into a 24‐well plate and stained with DCFH‐DA (10 μmol/L) for 20 min at 37°C. The ROS expression were observed and measured under a fluorescence microscope (Olympus).

### 2.9 Enzyme linked immunosorbent assay (ELISA)

The lung tissues were homogenized and lysed using a tissue homogenizer with radioimmunoprecipitation assay (RIPA), and the supernatant was obtained after centrifugation at 12,000 rpm for 10 min at 4°C. The levels of TNF‐α, IL‐1β, and IL‐6 were determined by ELISA kits (Beyotime) following the manufacturer's protocols. The absorbance was read by a microplate reader (Thermo Fisher) and the concentrations were shown in pg/mL.

### 2.10 Assessment of oxidative factors

The lung tissues were homogenized and lysed using a tissue homogenizer with RIPA, and the supernatant was obtained after centrifugation at 12,000 rpm at 4°C for 10 min. The contents of SOD, MDA, and GSHPx were analyzed by commercial diagnostic kits (Beyotime). The optical density was analyzed by a microplate reader (Thermo Fisher).

### 2.11 TdT‐mediated dUTP‐biotin nick end labeling (TUNEL) staining

The lung tissues were fixed with 4% paraformaldehyde, followed by embedded in paraffin and cut into 4 μm slices. Subsequently, the slices were dewaxed with xylene and rehydrated with reduced concentration ethanol. After that, the slices were covered with a solution of protease K for 20 min at room temperature. After washing with PBS for three times, the slices were incubated with the biotinylated terminal dUTP transferase and biotinylated dUTP in TdT buffer (30 mM Tris, pH 7.2, 140 mM sodium cacodylate, and 1 mM cobalt chloride) for 1 h at 37°C with terminal dUTP in the moisture chamber, followed by 4′,6‐diamidino‐2‐phenylindole (DAPI) (20 µg/mL) for 10 min at room temperature. The images were observed under the fluorescence microscopy (Olympus). The number of TUNEL positive cells = (number of TUNEL‐positive cells/number of all cells) × 100%.

### 2.12 Hematoxylin‐eosin (HE) staining

The right upper lobe of the rat lung was isolated, and then fixed with 4% formalin. Afterwards, the lung tissues were embedded in paraffin and then cut into slices (5 μm thickness). Afterwards, the slices were stained with hematoxylin solution for 5 min, followed by 0.5% eosin solution for 3 min. Then, the slices were dehydrated with gradient ethanol, transparentized with xylene, and mounted with resin. The morphological lesions of lung tissues were photographed under an optical microscope (Olympus). The lung injury score was calculated by evaluating the degree of inflammatory cell infiltration, hemorrhage, interstitial and alveolar edema, and the thickness of alveolar septum. 0 score = no damage; 1 score = mild damage; 2 score = moderate damage; 3 score = severe damage, 4 score = very severe damage. The score is the average of the total score of the five criteria.

### 2.13 Western blot assay

Proteins were extracted from the lung tissues using a tissue homogenizer with RIPA. The BCA Kit (Beyotime) was applied to determine the protein concentration. An equal amount of proteins were separated by sodium dodecyl sulfate‐polyacrylamide gel electropheresis and then transferred to polyvinylidene fluoride membranes. After blocked with 5% nonfat milk, the membranes were incubated with primary antibodies against phosphorylation‐IκBα (p‐IκBα) (#2859, 1:1000, Cell Signaling Technology), IκBα (#4812, 1:1000, Cell Signaling Technology), p‐p65 (ab76302, 1:1000, Abcam), p65 (ab16502, 1:1000, Abcam), and GAPDH (ab181602, 1:2000, Abcam) overnight at 4°C, followed by horseradish peroxidase‐conjugated goat anti‐rabbit IgG secondary antibody (ab205718, 1:2000, Abcam) at 37°C for 1 h. The protein blots were visualized with the enhanced chemiluminescence kit and the optical density was analyzed with ImageJ software (National Institute of Health, Bethesda).

### 2.14 Statistical analysis

All data were presented as mean ± standard deviation. SPSS 25.0 software (SPSS Inc.) was applied for statistical analysis. The statistical significance among multiple groups were analyzed using the one‐way analysis of variance with Turkey post hoc test. A *p* < .05 represented statistical significance.

## 3 RESULTS

### 3.1 PFD effectively alleviated lung injury in SILI rats

**Figure 1:**
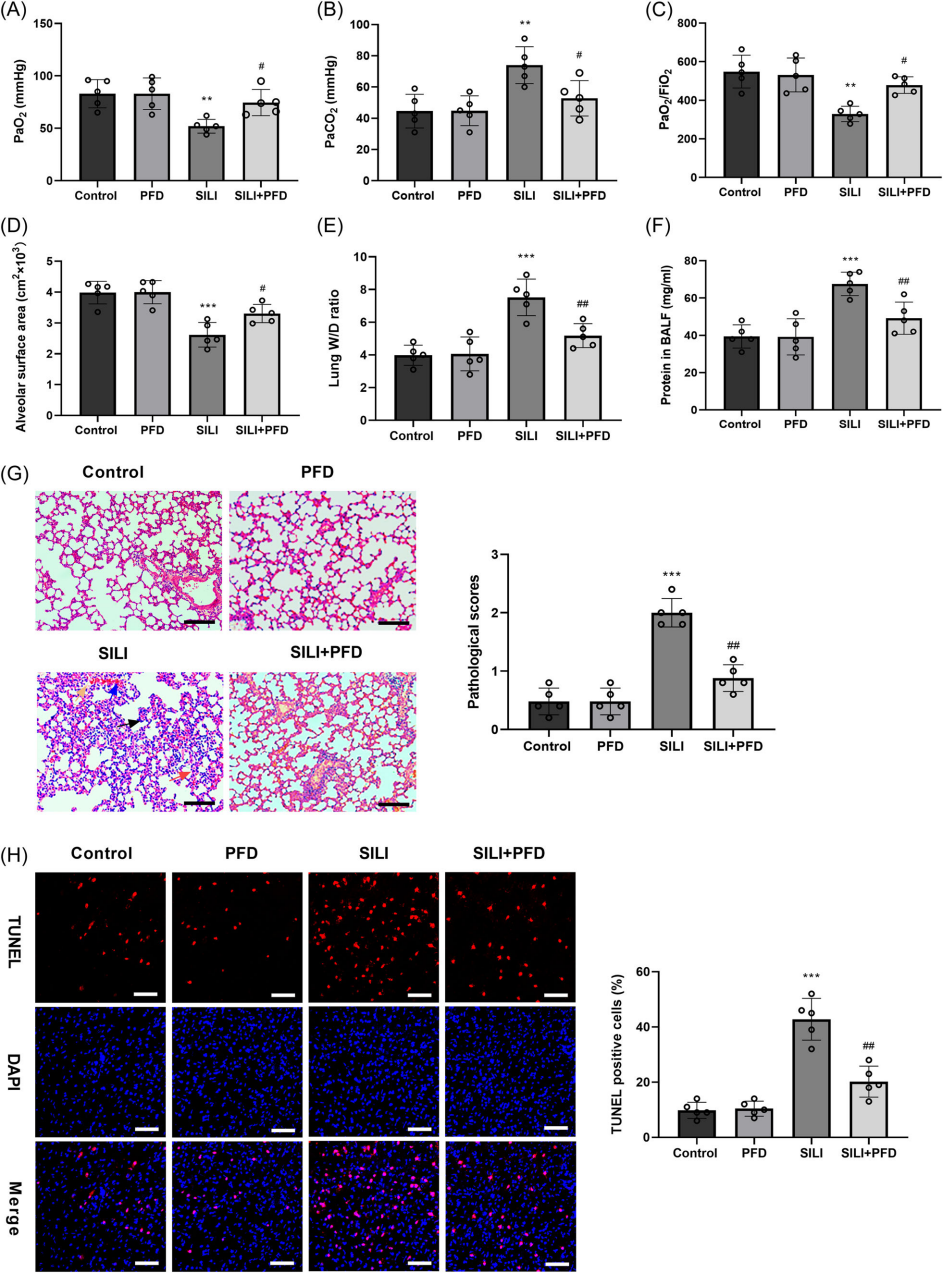
PFD effectively alleviated lung injury in SILI rats. (A) The values of PaO_2_, (B) PaCO_2_, and (C) PaO_2_/FiO_2_ in arterial blood, and (D) The alveolar surface area were measured to access the pulmonary function. (E) W/D lung weight ratio was detected to evaluate the lung edema. (F) The protein concentration in the BALF was assessed by BCA protein concentration assay kit. (G) HE staining was utilized for determining the interstitial and alveolar edema, inflammatory cell infiltration, the thickness of alveolar septum and diffuse hemorrhage in lung tissues (edema, orange arrow; inflammatory cell infiltration, black arrow; alveolar septum thickening, red arrow; diffuse hemorrhage, blue arrow) (scale bar = 50 μm). (H) TUNEL staining was utilized for determining the apoptosis in the lung tissues (scale bar = 25 μm). *N* = 5. Results are presented as means ± *SD*, analyzed using ANOVA, followed by Turkey post hoc test. **p < .01, ***p < .001 versus Control group; ^#^ *p* < .05, ^##^ *p* < .01 versus SILI group. ANOVA, analysis of variance; HE, hematoxylin‐eosin; PFD, pirfenidone; *SD*, standard deviation; SILI, smoke inhalation lung injury; TUNEL, TdT‐mediated dUTP‐biotin nick end labeling; W/D, wet:dry.

To assess the protective effects of PFD on SILI, we conducted an in vivo study using SILI rats. Blood gas analysis results discovered that the PaO_2_ and PaO_2_/FiO_2_ levels were markedly declined, while PaCO_2_ level was significantly elevated in SILI group compared with control group. Treatment with PFD significantly increased the reduced levels of PaO_2_ and PaO_2_/FiO_2_, and decreased the elevated level of PaCO_2_ (Figure 1A–C). Moreover, the alveolar surface area was significantly reduced in SILI group versus to control group. Following treatment with PFD, the alveolar surface area was obviously elevated (Figure 1D). W/D and HE staining were adopted to detect the lung edema and histopathological changes, respectively. As depicted in Figure 1D, the W/D lung weight ratio was obviously elevated in SILI group, compared to the control group. After treatment with PFD, the W/D lung weight ratio was significantly decreased (Figure 1E). Meanwhile, the protein concentration in the BALF was higher in SILI group than that in the control group. However, the protein concentration in the BALF was lower in the group that received PFD therapy compared to that in the SILI group (Figure 1F). Results from HE staining revealed that PFD treatment inhibited the increase in inflammatory cell infiltration, partial alveolar septum thickening and diffuse hemorrhage induced by smoke inhalation (Figure 1G). TUNEL staining showed that there was no obvious apoptosis in the lung tissues of the control group, but the apoptosis increased significantly in the SILI group. On the contrary, PFD treatment triggered remarkable repression in cell apoptosis induced by smoke inhalation (Figure 1H). Collectively, these findings demonstrate that PFD improves smoke inhalation‐evoked lung injury in rats.

### 3.2 PFD reduced the inflammation and oxidative stress responses in SILI rats

**Figure 2:**
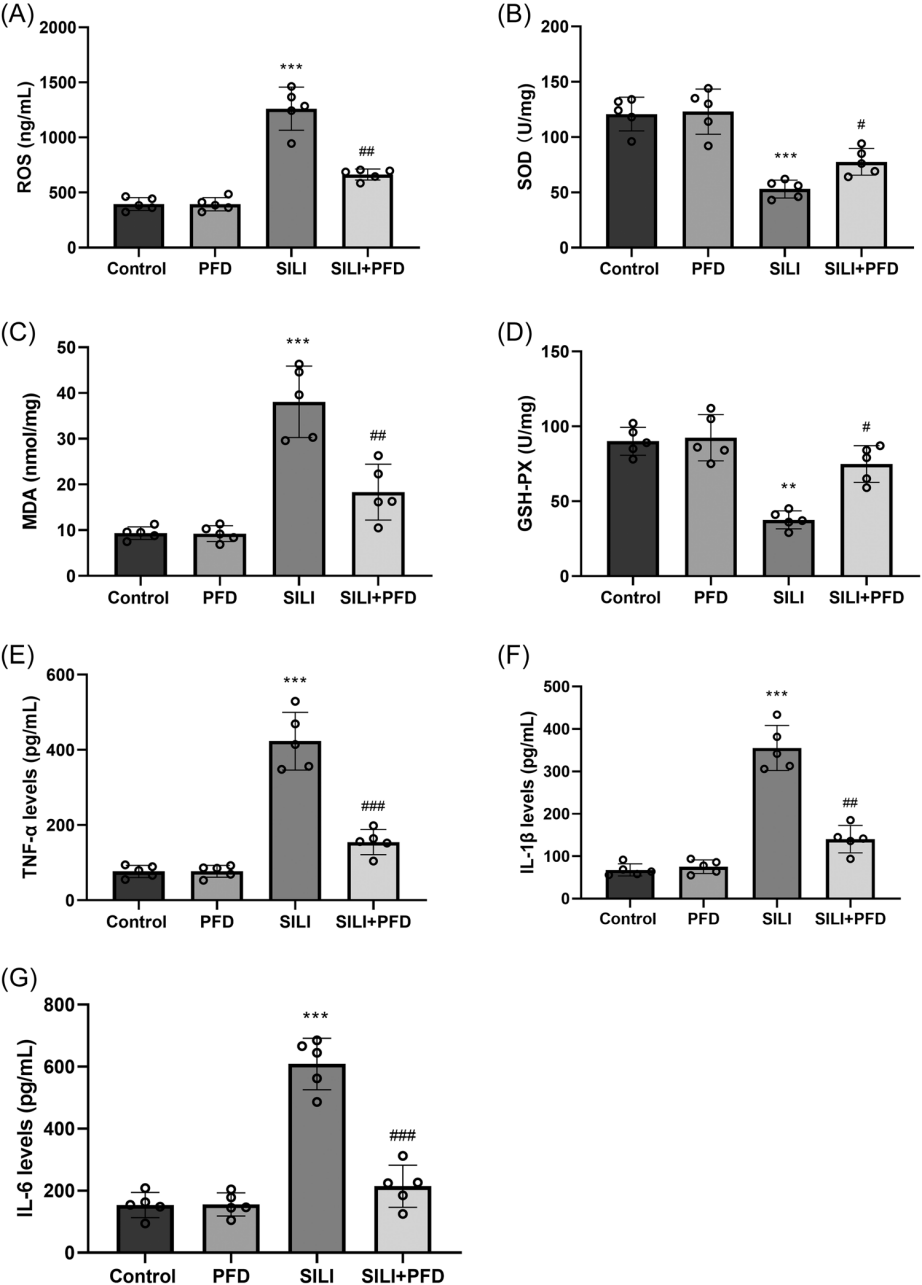
PFD reduced the inflammation and oxidative stress responses in SILI rats. (A) ROS level was determined by DCFH‐DA staining. (B–D) ELISA assay was adopted to determine the levels of oxidative stress related components (SOD, MDA, and GSH‐Px). (E–G) The release of inflammatory cytokines (TNF‐α, IL‐1β, and IL‐6) were accessed by ELISA assay. *N* = 5. Results are presented as means ± *SD*, analyzed using ANOVA, followed by Turkey post hoc test. **p < .01, ***p < .001 versus Control group; ^#^ *p* < .05, ^##^ *p* < .01, ^###^ *p* < .001 versus SILI group. ELISA, enzyme linked immunosorbent assay; IL, interleukin; MDA, malondialdehyde; PFD, pirfenidone; ROS, reactive oxygen species; SILI, smoke inhalation lung injury; TNF, tumor necrosis factor.

To evaluate the effects of PFD on oxidative stress response in SILI rats, ROS level was first determined in SILI rats after treatment with PFD. Results revealed that after smoke inhalation stimulation, there was a significant increase in ROS level, which was prevented via PFD treatment (Figure 2A). ELISA was subsequently carried out to determine the levels of oxidative stress related components. Results showed that the SOD and GSH‐Px levels in SILI group were decreased, while the MDA level was increased. However, following treatment with PFD, the levels of SOD and GSH‐Px were significantly increased, while the MDA level was significantly decreased (Figure 2B–D). We also analyzed the effects of PFD on smoke inhalation‐induced inflammatory injury. As presented by ELISA, the release of inflammatory cytokines, including IL‐1β, TNF‐α, and IL‐6 were remarkably elevated in SILI group, but PFD treatment inhibited these increases (Figure 2E–G). These above results confirm that PFD resists the inflammation and oxidative stress responses in SILI.

### 3.3 PFD suppressed the activation of NF‐κB pathway in SILI rats

**Figure 3:**
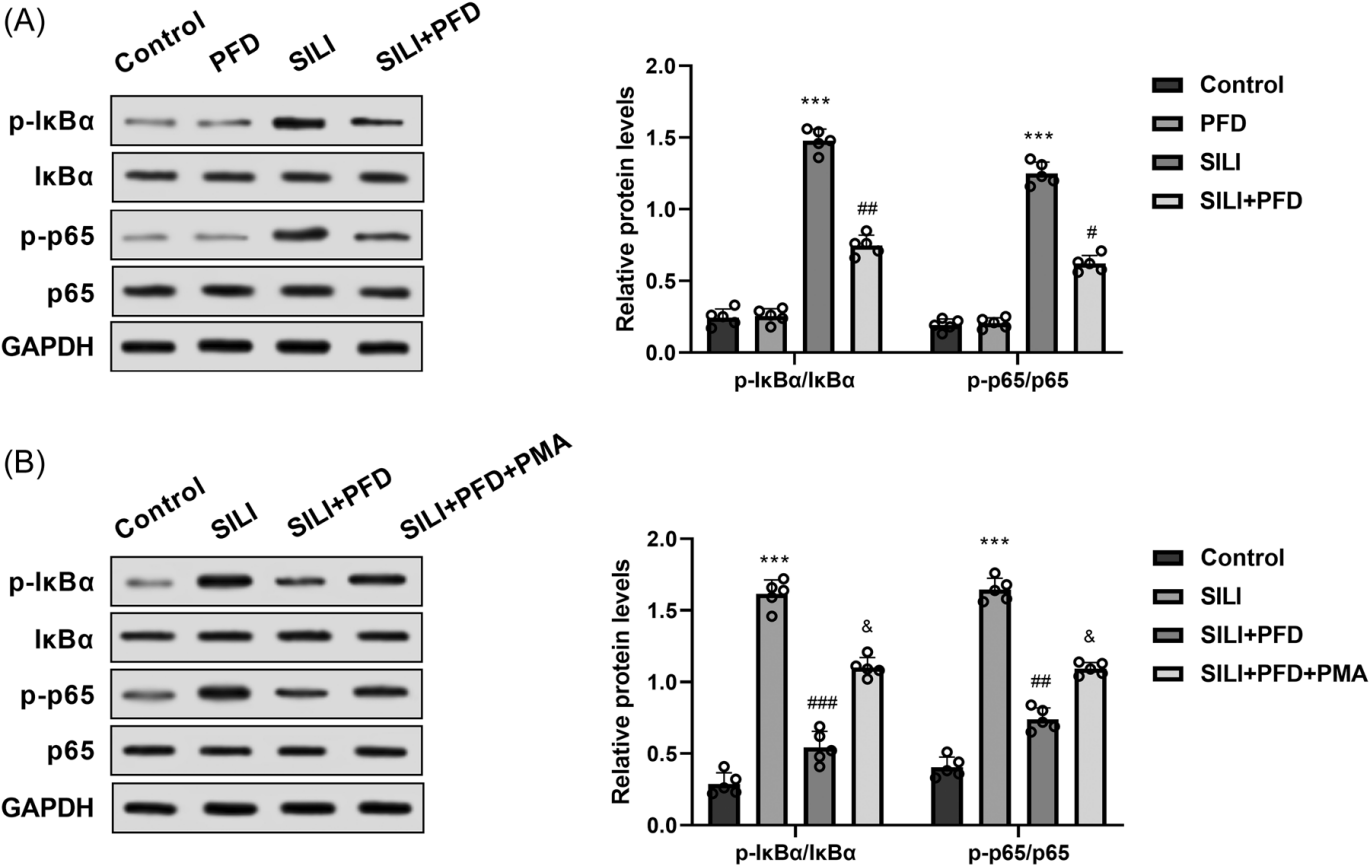
PFD suppressed the activation of NF‐κB pathway in SILI rats. (A) Western blot was adopted to elucidate the levels of p‐IκBα and p‐p65 in lung tissues of SILI rats. (B) Western blot was adopted to elucidate the levels of p‐IκBα and p‐p65 in lung tissues of SILI rats after addition of NF‐κB agonist PMA. *N* = 5. Results are presented as means ± *SD*, analyzed using ANOVA, followed by Turkey post hoc test. ***p < .001 versus Control group; ^##^ *p* < .01, ^###^ *p* < .001 versus SILI group; ^&^ *p* < .05 versus SILI + PFD group. ANOVA, analysis of variance; NF‐κB, nuclear factor kappa B; *SD*, standard deviation; SILI, smoke inhalation lung injury.

To elucidate the potential pathway by which PFD prevented SILI, western blot was utilized to measure the related protein levels of NF‐κB pathway. Results discovered that the p‐IκBα/IκBα and p‐p65/p65 ratios were markedly increased in the rats exposed to smoke inhalation, while administration of PFD suppressed these elevated levels (Figure 3A). PMA, NF‐κB agonist, was then used for activating NF‐κB pathway. Results showed that addition of PMA reversed PFD‐mediated inhibition of NF‐κB signaling pathway in SILI rats (Figure 3B). These findings illustrate that PFD inhibited the activation of NF‐κB signaling pathway in SILI rats.

### 3.4 NF‑κB signaling activation reversed the effects of PFD on SILI in rats

**Figure 4:**
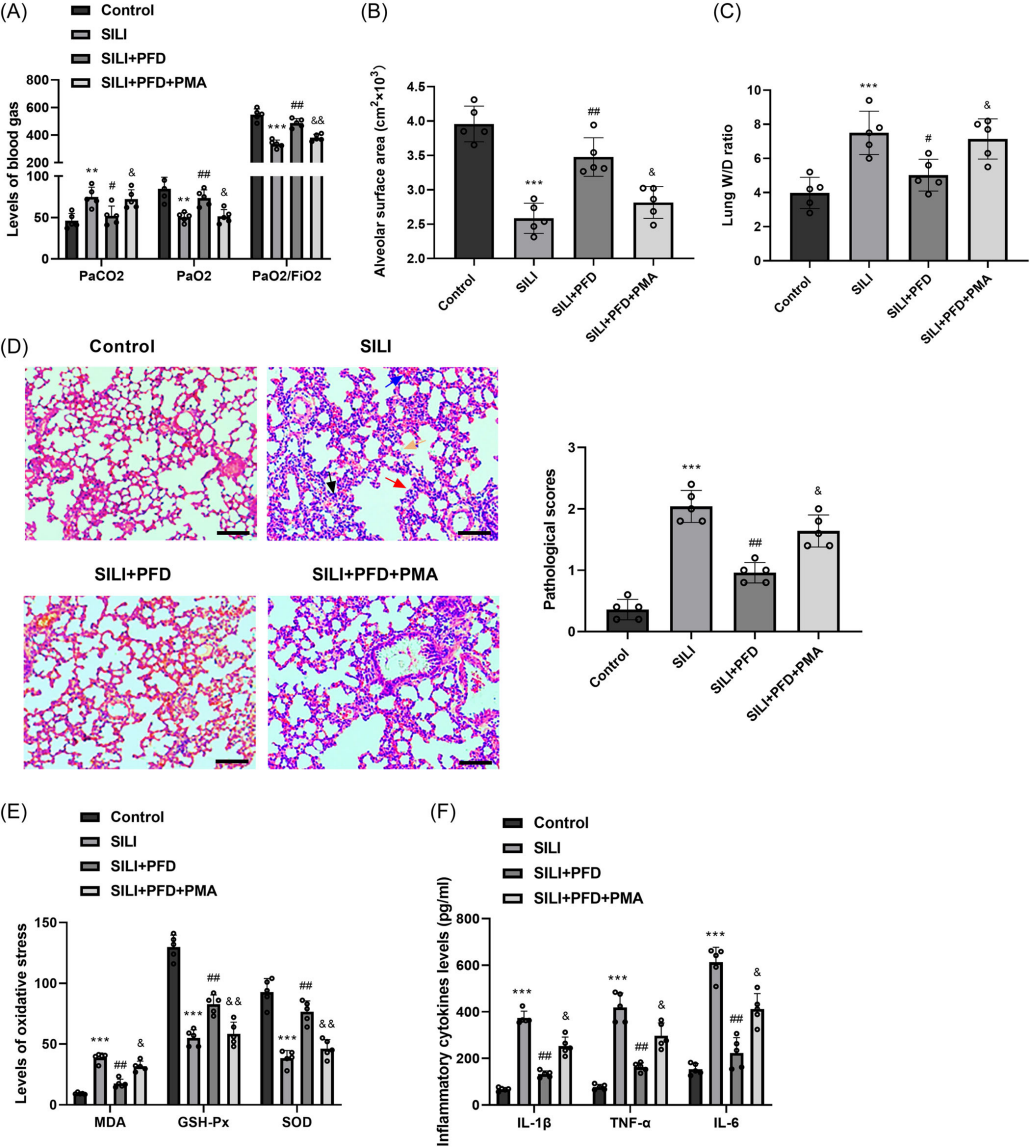
NF‑κB signaling activation reversed the effects of PFD on SILI in rats. (A) The values of PaO_2_, PaCO_2_, and PaO_2_/FiO_2_ in arterial blood were measured in rats after treatment with PMA. (B) The alveolar surface area was measured in rats after treatment with PMA. (C) W/D lung weight ratio was detected in rats after treatment with PMA. (D) HE staining was utilized for determining the interstitial and alveolar edema, inflammatory cell infiltration, the thickness of alveolar septum and diffuse hemorrhage of lung tissues of rats after treatment with PMA (edema, orange arrow; inflammatory cell infiltration, black arrow; alveolar septum thickening, red arrow; diffuse hemorrhage, blue arrow) (scale bar = 50 μm). (E) ELISA analysis was adopted for determining the oxidative stress and (F) inflammation in the lung tissues of rats after treatment with PMA. *N* = 5. Results are presented as means ± *SD*, analyzed using ANOVA, followed by Turkey post hoc test. **p < .01, ***p < .001 versus Control group; ^#^ *p* < .05, ^##^ *p* < .01 versus SILI group; ^&^ *p* < .05, ^& &^ *p* < .01 versus SILI + PFD group. ANOVA, analysis of variance; ELISA, enzyme linked immunosorbent assay; HE, hematoxylin‐eosin; NF‐κB, nuclear factor kappa B; *SD*, standard deviation; SILI, smoke inhalation lung injury; W/D, wet:dry.

To investigate whether or not PFD requires the NF‐κB pathway to protect against SILI, we analyzed the effects of addition of PMA, the agonist of NF‑κB signaling pathway, on SILI. As depicted in Figure 4A, after activation of NF‐κB pathway by PMA, PaCO_2_ level was elevated whereas PaO_2_ and PaO_2_/FiO_2_ levels were declined in the blood, compared to SILI + PFD group. Moreover, the addition of PMA partially reversed the protective effect of PFD on SILI, as shown by a decrease in alveolar surface area, an increase in the W/D ratio, and a decrease in inflammatory cell infiltration, partial alveolar septum thickening and diffuse hemorrhage of lung tissues (Figure 4B–D). In addition, PMA mitigated the decrease of MDA level and reduced the increase of GSH‐Px and SOD levels mediated by PFD (Figure 4E). Meanwhile, results from ELISA showed that PMA restored the inhibition effects of PFD on smoke inhalation‐induced inflammation in the lung tissues of rats (Figure 4F). These results confirm that PFD resists SILI through the NF‐κB pathway.

## 4 DISCUSSION

SILI refers to the damage of upper respiratory tract, tracheobronchus and lung parenchyma caused by smoke inhalation. The chemical irritants in smoke are particles and gases of varying sizes, which lead to progressive cell damage and severe lung injury that, if not diagnosed and treated promptly, it may progress to acute respiratory distress syndrome, eventually leading to multiple organ dysfunction syndrome and even death.^25^, ^26^ Previous studies have shown that irritation of the respiratory system with a range of harmful chemicals, such as carbon monoxide, cyanide, and malondialdehyde, leads to breathing difficulties and even asphyxia, which is associated with oxidative stress/antioxidant stress imbalances and an inflammatory cascade.^27^, ^28^ In this paper, we demonstrated for the first time the protective effects of PFD against SILI. Moreover, PFD was found to protect against SILI via inhibiting inflammation, oxidative stress, and apoptosis. Mechanistically, PFD alleviated SILI via the NF‐κB signaling pathway in rats.

Studies on the mechanisms suggest that in the early stage of SILI, smoke particles stimulate the body's inflammatory cells to release a large number of inflammatory cytokines such as IL‐1β, IL‐6, TNF‐α, resulting in increased vascular permeability and pulmonary edema. At the same time, the increase of inflammatory cytokines leads to the massive production of nitric oxide (NO) and ROS in respiratory endothelial cells and alveolar cells, further enhancing oxidative stress damage, and the continuous imbalance of oxidative stress leads to cell death.^29^, ^30^ Therefore, inhibition of lung inflammation and oxidative stress after smoke inhalation is the main measure to deal with SILI. A previous study has shown that the underlying mechanism of PFD on idiopathic pulmonary fibrosis may be related to inhibiting inflammation and alleviating oxidative stress.^31^ Another study displayed that PFD treatment significantly improved the symptoms of pulmonary edema and hypoxemia through inhibiting ROS‐mediated inflammasome activation.^32^ The study of Tsuchiya et al.^23^ showed that PFD inhibited the production of oxygen free radicals, thereby inhibiting inflammatory cytokines such as IL‐6 and TNF‐α, and improving pulmonary edema.^33^ In this study, we constructed a SILI model in rats to assess the protective effects of PFD on SILI, and results demonstrated that PFD treatment significantly improves the SILI‐induced respiratory function decline and pathological injuries, such as pulmonary edema, thickening of alveolar wall, inflammatory cell infiltration, and diffuse bleeding. In addition, PFD treatment inhibited the release of inflammatory cytokines (IL‐1β, TNF‐α, and IL‐6), moreover, following treatment with PFD, the levels of the SOD and GSH‐Px were increased, and the ROS and MDA levels were decreased. Furthermore, PFD treatment triggered remarkable repression in cell apoptosis induced by smoke inhalation. These results suggest that PFD prevents severe SILI through inhibiting inflammation, oxidative damage, and apoptosis.

Identifying PFD‐associated signaling pathways in SILI that regulate inflammation and oxidative stress to ameliorate lung injury is critical to elucidating the pathogenesis of SILI. NF‐κB has been shown to be an important pathway in the modulation of inflammation and oxidative stress responses, which contributes to acute lung injury.^34^, ^35^, ^36^ For instance, Liu et al.^37^ demonstrated that Xuanfei Baidu Formula inhibited acute lung injury induced by LPS via suppressing the NF‐κB signaling pathway. It has shown by Chen et al.^38^ that NF‐κB aggravates acute lung injury in mice via miR‐99/PRDM1 axis. Moreover, a study indicated that Dapk1 alleviated the oxidative stress and inflammation in LPS‐induced acute lung injury via repressing p38MAPK/NF‐κB pathway.^39^ It is worth noting that the inactivation of NF‐κB is key to prevent smoke inhalation‐induced lung injury.^24^ Fortunately, our results discovered that the NF‐κB signaling pathway was activated in SILI rats and NF‐κB activation significantly reversed the suppression effects of PFD on inflammation and oxidative damage in SILI rats.

However, several limitations were also remained. First, we only evaluated the protective effects of PFD on SILI in vivo, while its role in lung cells and immune cells was poorly explored. Second, this study only testified the efficacy of PFD in the SILI animal model, and its effect on SILI patients should verified in the future. Third, some studies have suggested that lung damage caused by smoking showed a sex difference, but in this study, we only investigated the protective effect of PFD on SILI in male rats, and its effect on SILI in female rats needs to be further verified. Moreover, the therapeutic effects of PFD on the survival of SILI patients should be further studied on the basis of previous experiments to explore the more detailed pathological mechanism.

## 5 CONCLUSIONS

PFD alleviated lung tissue damage, inflammation, and oxidative stress in SILI rats, potentially via repressing the NF‐κB pathway. This research proposed the underlying mechanism of PFD against SILI, indicating that PFD may be used for SILI therapy.

## AUTHOR CONTRIBUTIONS

Jinxiang Wang designed and supervised the research. Tingting Lv performed the research and draft the manuscript. Kaiyuan Yang performed data analysis and statistical analysis. Both authors read and approved the final manuscript. Both authors have participated sufficiently in the work and agreed to be accountable for all aspects of the work.

## CONFLICT OF INTEREST STATEMENT

The authors declare no conflicts of interest.

## ETHICS STATEMENT

The study was approved by the Ethics Committee of Capital Medical University (No. AEEI‐2023‐156).

## Data Availability

The datasets used and/or analyzed during the present study are available from the corresponding author on reasonable request.
